# Expert Consensus to Explore the Definition and Characterization of Methamphetamine-Associated Pulmonary Arterial Hypertension and Key Treatment Considerations

**DOI:** 10.1016/j.chpulm.2025.100232

**Published:** 2025-12-31

**Authors:** Roham Zamanian, Peter J. Leary, Therese Sargent, John Kingrey, David Lopez, Ankita D. Adhia, Gurinderpal Doad, Marinella Sandros, Medi Stone, Daisy Bridge, Richard Perry, Ashley Enstone, Lisa Perrett, Holly Smith, Nick H. Kim

**Affiliations:** a Pulmonary and Critical Care, Stanford University School of Medicine, Stanford, CA; b University of Washington Medicine, Seattle, WA; c Honor Health Pulmonary Hypertension Clinic, Phoenix, AZ; d INTEGRIS Health, Oklahoma City, OK; e Johnson & Johnson, Titusville, NJ; f Adelphi Values PROVE, Bollington, Cheshire, England; g University of California San Diego, San Diego, CA

**Keywords:** combination therapy, methamphetamine, methamphetamine-associated pulmonary arterial hypertension, pulmonary arterial hypertension, treatment considerations

## Abstract

**Background:**

Methamphetamine use can result in methamphetamine-associated pulmonary arterial hypertension (Meth-APAH). Compared with idiopathic pulmonary arterial hypertension (PAH), Meth-APAH may have a worse clinical course.

**Research Question:**

How can methamphetamine use status be classified and what are the associated treatment considerations for patients with Meth-APAH?

**Study Design and Methods:**

US-based experts (N = 12) who manage patients with Meth-APAH were recruited to a double-blinded, modified Delphi panel (2 survey rounds and a virtual consensus meeting). During the survey rounds, panelists answered a range of questions with free-text responses or rating statements. For rating questions, consensus was defined as ≥ 80% of panelists rating their agreement (from 7 to 9) or disagreement (from 1 to 3) using a 9-point Likert scale.

**Results:**

The panel was unable to reach consensus on the definition of Meth-APAH. Although there was general agreement that Meth-APAH is a group 1 PAH diagnosis in a patient with a history of methamphetamine use, a number of panelists thought that additional details were needed (eg, duration, cumulative exposure) and these details could not be agreed on. The panel agreed that Meth-APAH is heterogeneous in disease course and physicians should explore the potential of methamphetamine use in all new and existing cases of PAH regardless of the regional prevalence of methamphetamine use. The panel achieved consensus that patients with Meth-APAH should be treated for both PAH and drug addiction. There are no Meth-APAH-specific barriers to double combination therapy (ie, endothelin receptor antagonists, phosphodiesterase 5 inhibitors), but a range of patient and disease factors may influence the choice and route of delivery for other therapies.

**Interpretation:**

The characterization of Meth-APAH may help facilitate the identification, treatment, and management of patients with Meth-APAH to improve clinical outcomes.

**Take-Home Points**

**Study Question:** How can methamphetamine use status be classified and what are the associated treatment considerations for patients with methamphetamine-associated pulmonary arterial hypertension (Meth-APAH)?

**Results:** The panel discussed that Meth-APAH can be defined as follows: group 1 pulmonary arterial hypertension in a patient with history of methamphetamine use; however, consensus was not achieved. Also, the required exposure, duration, dose, and frequency of methamphetamine use that is likely to cause Meth-APAH failed to reach consensus.

**Interpretation:** The characterization of Meth-APAH may help facilitate the identification, treatment, and management of patients with Meth-APAH to improve clinical outcomes.

Pulmonary arterial hypertension (PAH) is a progressive and rare disease of the pulmonary vasculature with increasing global incidence.^1^ In addition to idiopathic PAH (IPAH), heritable PAH, and other related conditions (eg, connective tissue disease, HIV), drugs and toxins have also been associated with the development of PAH.^2^ Due to phenotypic similarities with IPAH, drug-and-toxin-induced PAH is included in the World Health Organization (WHO) group I classification.^3^

One of the first compounds that was found to have an association with PAH was rapeseed oil, which led to an acute epidemic of toxic oil syndrome in Spain in the 1980s.^4^^,^^5^ Fenfluramine/phentermine, which was previously used as an anorectic drug for those with obesity, was later documented to also be associated with PAH and linked to the deaths of otherwise healthy individuals.^6^^,^^7^ Some cancer medications have also been found to have definite association with PAH, including dasatinib, mitomycin-C, and carfilzomib.^8^^,^^9^ Between the 2015 and 2018 European Society of Cardiology and European Respiratory Society Guidelines, methamphetamine use has changed from likely being associated with methamphetamine-associated PAH (Meth-APAH) to definitely associated.^3^ Methamphetamine is a highly addictive derivative of amphetamine and was initially developed as a medication for several neuropsychiatric conditions, including attention-deficit hyperactivity disorder.^10^

Methamphetamine use is a growing global health concern, with particularly high prevalence in the West and Midwest regions of the United States.^10^, ^11^, ^12^ Among specialty PH centers, where PAH is most likely to be adequately evaluated and appropriately diagnosed, the prevalence of Meth-APAH among all patients with any type of PAH is estimated to be 9% (as shown through the Pulmonary Hypertension Association Registry cohort).^13^ There is currently no consensus on the definition/classification of Meth-APAH and its treatment regimens, which could result in underdiagnosis and suboptimal management.^3^^,^^14^

Previous cohort studies have suggested differences in the clinical characteristics and outcomes for patients with Meth-APAH vs IPAH. A 2018 single-center prospective study reported that patients with Meth-APAH (n = 90) had more severe pulmonary vascular disease and a worse prognosis than patients with IPAH (n = 97). The risk of clinical worsening or death was also double that of IPAH (hazard ratio, 2.04; 95% CI, 1.28-3.25; *P* = .003).^15^ Additionally, a multicenter registry prospective study reported that patients with Meth-APAH (n = 118) were generally younger, and had lower socioeconomic status (SES), levels of education, income, and employment rates than patients with IPAH (n = 423).^13^ These social and psychological factors may present further challenges in managing patients with Meth-APAH.^16^

Understanding patient history and status of methamphetamine use is hypothesized to be vital in understanding disease management and prognosis. However, due to the stigma associated with methamphetamine use, many patients are reluctant to disclose information to clinicians.^14^ In addition, methamphetamine use is highly comorbid with mental illness, other substance abuse, and low health literacy, as reported by Xu and Zhao.^16^ These factors pose unique challenges to care delivery.^16^

According to the 2022 European Society of Cardiology/European Respiratory Society Guidelines for the treatment of PAH, the treatment algorithm for Meth-APAH is not distinguished from IPAH.^17^ Current management approaches for Meth-APAH are variable but may include routine drug screening, sessions with a social worker, drug rehabilitation, and oral PAH-specific therapy.^13^ A prospective study by Malhotra et al^18^ found that patients with Meth-APAH generally show a less robust response to inhaled nitric oxide, predicting a diminished response to certain therapeutic agents (ie, calcium channel blockers). Furthermore, clinical teams are reluctant to use parenteral prostacyclin analogs in patients with Meth-APAH given concerns regarding appropriate central line/skin site care and safety.^15^ In addition, lower proportions of patients specifically identified as having Meth-APAH have been enrolled in clinical studies to date, further widening knowledge gaps in this population.^16^

The Delphi panel methodology is an established approach for developing and recording consensus on areas where evidence is low. Gathering expert opinion using the Delphi method on the classification of methamphetamine use status and associated treatment considerations in patients with Meth-APAH may be used to inform future treatment paradigms.

## Study Design and Methods

### Modified Delphi Panel and Panelists

**Figure 1:**
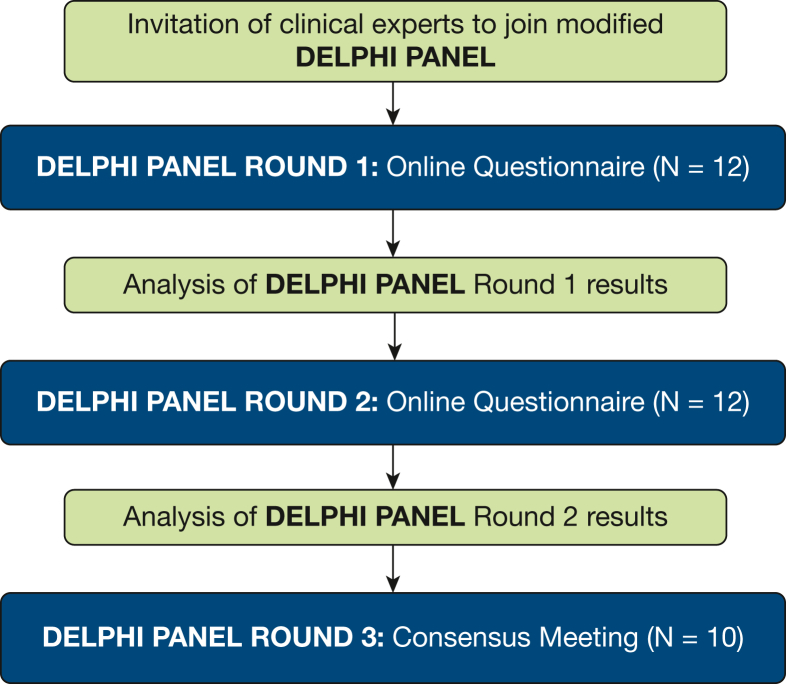
Overview of the modified Delphi methodology.

A double-anonymized modified Delphi method was used to investigate the classification of methamphetamine use status and associated treatment considerations for patients with Meth-APAH.^19^ The modified Delphi panel included 2 survey rounds followed by a final virtual consensus meeting (Fig 1).

US expert physicians who actively manage patients with Meth-APAH were approached by a third-party agency, and 12 panelists were recruited based on prespecified screening criteria. The panel size (N = 12) was sufficiently large to draw robust conclusions and reach consensus on different aspects of Meth-APAH.^20^, ^21^, ^22^ Management of panelists via the third-party agency allowed for the maintenance of study integrity, panelist anonymity, and minimized bias. Key screening criteria are summarized in Table 1.

**Table 1:** Summary of Panelist Characteristics

Criteria	Responses	
Specialty area	Cardiology	1
	Pulmonology	11
Years in practice	3-4	1
	5-10	4
	11-30	5
	> 30	2
Mean No. of patients with PAH in a 3-mo period	Mean [SD]	57 [29]
	Minimum	5
	Maximum	120
% of patients with PAH that have Meth-APAH	1-10	6
	11-20	1
	21-30	2
	31-40	3
US state	California	4
	Arizona	1
	Florida	1
	Indiana	1
	Massachusetts	1
	New York	1
	Oregon	1
	Texas	1
	Utah	1
Type of practice	CCC	6
	AMC	5
	Private practice	1

Data are presented as No. or as otherwise indicated. AMC = academic medical center; CCC = Center of Comprehensive Care; Meth-APAH = methamphetamine-associated pulmonary arterial hypertension; PAH = pulmonary arterial hypertension; US = United States.

### Ethical Compliance

This article is based solely on the opinions of a group of experts. Therefore, no patient data or information were collected and there was no requirement to obtain informed consent. Because expert consensus falls outside the remit of the governance arrangements for research ethics committees, ethical committee approval was not required. However, the study was conducted in accordance with ethical principles that have their origin in the Declaration of Helsinki and that are consistent with Good Pharmacoepidemiology Practices and applicable laws and regulations of the United States, as appropriate. The study remained compliant with the General Data Protection Regulation.

### Data Collection and Analysis

The surveys were programmed using an online platform and distributed to panelists through the third-party agency using email links. Sequential recruitment was used and physicians who met the screening criteria recruited until quotas were filled. Data were extracted into and analyzed in Microsoft Excel (Microsoft Inc). Key topics included the classification and definition of Meth-APAH, potential areas for improvement when considering existing care practices, treatment implications associated with methamphetamine use, and challenges with clinical data to support treatment options for patients with Meth-APAH.

**Figure 2:**
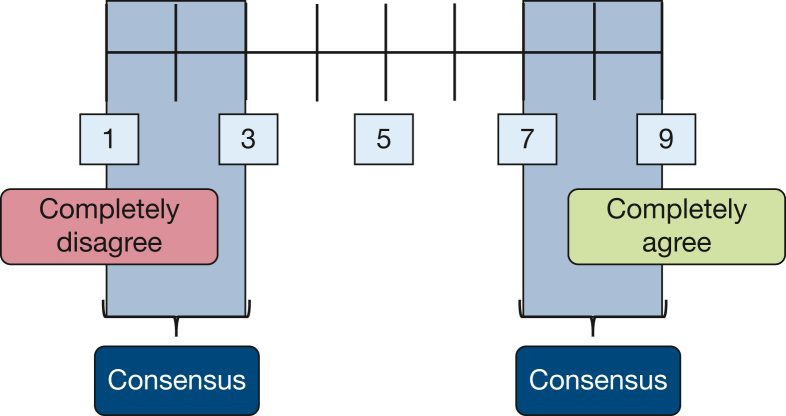
Likert scale used to assess consensus.

The first-round survey comprised open-ended questions requiring qualitative responses and statements for panelists to rate based on a 9-point Likert scale (Fig 2). The definition of consensus for this study was ≥ 80% of the participants agreed (or disagreed) with a statement on the 1 to 9 Likert scale, whereby agreement was represented as a rating between 7 and 9 and disagreement was represented as a rating between 1 and 3. The term consensus was used to refer to consensus in agreement throughout this article unless otherwise specified. The second round explored topics nearing consensus and those identified via free-text responses, in addition to identifying further topics for exploration in the consensus meeting. For areas not reaching consensus in the first round, the group’s mean response was presented alongside the panelist’s individual response. Any questions that reached consensus during the first round were not investigated further.

After completion of the survey rounds, a 3-hour virtual consensus meeting was held in December 2023. The data outputs of the survey rounds were summarized and presented in Microsoft PowerPoint (Microsoft Inc), including statements and questions to pose to the panel. This meeting facilitated discussion on the statements that did not reach consensus during the survey rounds and encouraged panelists to alter statements where appropriate (eg, syntax change) to reach consensus or provide rationale as to why consensus could not be met. Scientific Advisory Committee members who are named authors on this paper did not participate in this panel nor influence the votes of panel members.

## Results

### Summary of Results

Twelve physicians were recruited (Table 1), all of whom completed the first and second round surveys. Ten panelists participated in the final consensus meeting due to availability (Fig 1). Overall, 112 statements reached consensus (e-Table 1).

### Definition of Meth-APAH

The panel was unable to reach consensus on the definition of Meth-APAH. Although there was general agreement that Meth-APAH is a group 1 PAH diagnosis in a patient with a history of methamphetamine use, a number of panelists thought that additional details were needed (eg, duration, cumulative exposure), and these details could not be agreed on. The heterogeneity of the Meth-APAH patient population also impacts the definition and characterization of Meth-APAH.

Consensus was achieved that patients with Meth-APAH can be of any SES, and that SES should not be used to characterize patients because this can reinforce negative stereotypes that can lead to health care provider bias and the misconception that only disadvantaged patients should be screened for drug use.

The panelists achieved consensus that methamphetamine use type (particularly current use) impacts compliance to therapy, treatment escalation, and access to a range of therapies, and that current use (within the last month) impacts successful Meth-APAH outcomes. The panel also reached consensus that the other methamphetamine use types are not well established and lack precision. For example, the lengths of time to characterize recent or long-term methamphetamine abstinence were not well defined.

### Classification of Meth-APAH

**Figure 3:**
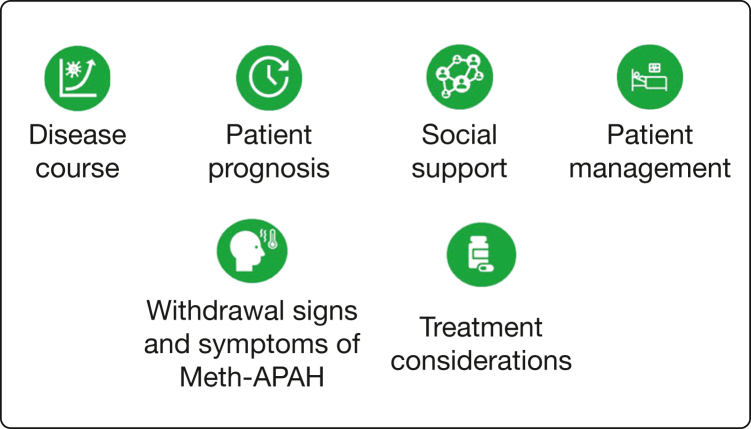
Factors that achieved consensus on differentiating Meth-APAH from idiopathic pulmonary arterial hypertension. Meth-APAH = methamphetamine-associated pulmonary arterial hypertension.

The panel reached consensus on several factors that could differentiate Meth-APAH from IPAH (Fig 3). However, there was a lack of consensus that symptoms were more severe in patients with Meth-APAH than in IPAH. Some panelists expressed concern that patterns of care for a subset of patients that sought care only at their “sickest,” and who then exhibit high utilization of inpatient and emergency care, may impact the perception of symptom severity in Meth-APAH.

Panelists did not collectively agree that the clinical presentation of Meth-APAH systematically differed from IPAH. The overall heterogeneity of the patient population with Meth-APAH was highlighted by panelists. Panelists also noted that although Meth-APAH varies in disease course, methamphetamine cessation likely has a positive impact on disease course and may lead to hemodynamic improvements.

There was also a lack of consensus among the panelists that the following factors would be suggestive of Meth-APAH: weight/muscle loss, obesity, dyspnea, or withdrawal symptoms; these factors were regarded as being too nonspecific, (eg, they could be due to withdrawal from other drugs) and did not reliably distinguish between participants with a range of PAH etiologies.

### Diagnosis of Meth-APAH

The panel achieved consensus that methamphetamine use is inquired about/explored for all cases of PAH, and that all patients presenting with PAH should be screened for Meth-APAH. Furthermore, the discussion of drug history and previous methamphetamine use are assessments that distinguish between patients with different forms of PAH.

There was no consensus on when patients should be screened for methamphetamine use before right heart catheterization. However, consensus was reached that physicians would still consider right heart catheterization in a patient who is a known to have active methamphetamine use. The panel noted disparity between what is ideal and what is practical when managing some patients.

Patient-related, treatment-related, and socioeconomic barriers to identifying patients with Meth-APAH exist and are summarized in Table 2. Additionally, a lack of clinician familiarity with Meth-APAH is a barrier to identifying patients because it can limit appropriate engagement or screening. Regional differences were highlighted wherein physicians who practice in geographic areas where methamphetamine use/Meth-APAH is more prevalent will be more familiar with Meth-APAH than those in regions with lower prevalence.

**Table 2:** Barriers That Achieved Consensus to Identifying Patients With Meth-APAH

**Patient-Related Factors**
• Lack of familial and caregiver support (access to care) • Patient engagement with care (access to care)
**Treatment-Related Factors**
• Clinician familiarity with Meth-APAH (lack of recognition) • Left-sided heart disease (lack of recognition in the setting of medical complexity) • HIV (lack of recognition in the setting of medical complexity)
**Socioeconomic Factors**
• Access to transportation (access to care) • Lack of stable housing (access to care) • Socioeconomic status (access to care)

Meth-APAH = methamphetamine-associated pulmonary arterial hypertension.

### Treatment Implications for Meth-APAH

Disease-, patient-, and treatment-related factors affecting treatment choice in Meth-APAH that achieved consensus are summarized in Table 3. Consensus was reached that patients who actively use methamphetamine can and should be treated with established PAH combination therapies. Furthermore, methamphetamine use cessation should be among the main treatment goals for patients with Meth-APAH. However, there was a lack of consensus on whether cessation of methamphetamine is sufficient to prevent disease progression in patients with Meth-APAH, with 1 panelist noting that it is not because patients require pharmaceutical interventions. Thus, patients should still be treated with PAH therapies, in addition to receiving addiction support to achieve methamphetamine cessation.

**Table 3:** Disease-, Patient-, and Treatment-Related Factors That Achieved Consensus on Affecting Treatment Choice in Meth-APAH

Disease-Related Factors	Patient-Related Factors	Treatment-Related Factors
• Comorbidities • Presence of right-sided heart failure • Vasoreactivity response • Symptom severity at diagnosis • REVEAL risk score • Risk assessment or risk stratification	• Methamphetamine use status • Patient’s compliance with health care team • Ability to communicate/interact with the health care team • Patient’s social/caregiver support/friends/family • Stable housing/stable employment • Mental health/psychiatric conditions • Patient’s treatment adherence history • Participation in a substance abuse program	• Congenital heart disease • HIV

Meth-APAH = methamphetamine-associated pulmonary arterial hypertension; REVEAL = Registry to Evaluate Early and Long-Term PAH Disease Management.

Consensus was reached that there are no Meth-APAH-specific barriers to use of double-oral combination therapy (ie, endothelin receptor antagonists, phosphodiesterase 5 inhibitors). The panel felt that prescription of IV or subcutaneous (SC) therapy may require additional consideration for patients with Meth-APAH. The panel agreed that the prescription of IV/SC therapy should be dependent on safety, compliance, and practicality rather than methamphetamine use status. Panelists reached consensus that they are equally likely to prescribe injection therapies to a patient with Meth-APAH when they are compliant to therapy and have achieved sobriety compared with other forms of PAH. The panel thought that ongoing IV methamphetamine use, although rare, may be a prohibitive barrier to IV/SC prostacyclin analogs.

**Figure 4:**
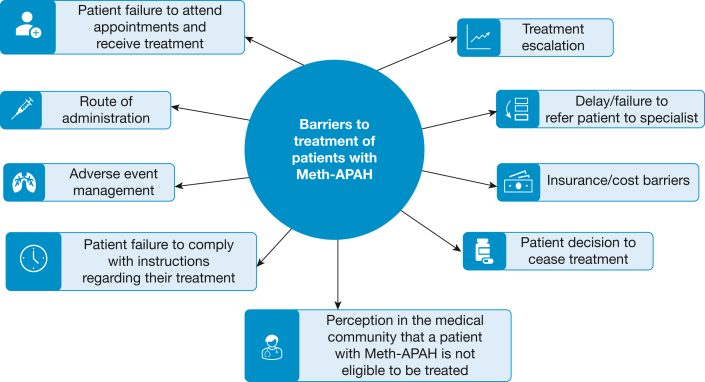
Barriers that achieved consensus to treatment of patients with Meth-APAH. Meth-APAH = methamphetamine-associated pulmonary arterial hypertension.

Factors that achieved consensus as barriers to the treatment of patients with Meth-APAH are summarized in Figure 4. Broadly, the panel agreed that key barriers to treatment included lack of recognition or access at multiple levels. Notably, these barriers may not be specific to Meth-APAH and may impact barriers to treatment in many forms of PAH.

**Figure 5:**
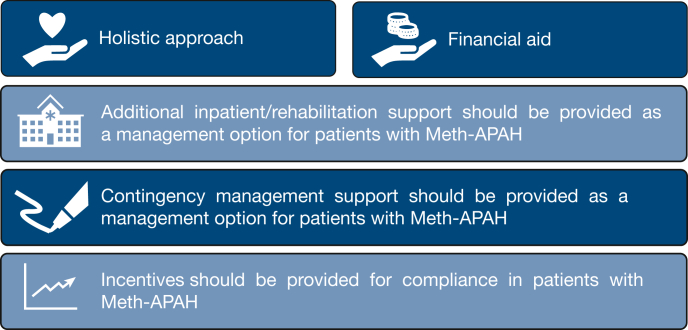
Support resources and strategies that achieved consensus for improving patient management and manage nonadherence to treatment in Meth-APAH. Meth-APAH = methamphetamine-associated pulmonary arterial hypertension.

The panel also achieved consensus that loss to follow-up is a key challenge to managing patients with Meth-APAH. Support resources and strategies to improve the management of patients with Meth-APAH that achieved consensus are summarized in Figure 5. The panel achieved consensus that methamphetamine cessation was a key factor in patient management and might include additional inpatient/rehabilitation support, contingency management support, and incentives for patients with Meth-APAH. In addition, the panel agreed that approaches to encourage persistence with therapy, including mitigation of insurance barriers and adverse event management, were valuable for patients with Meth-APAH, and physicians agreed that they would be likely to prescribe oral medications that reduce pill count.

The panel achieved consensus that patients with Meth-APAH should be included in clinical trials to generate information regarding treatment and disease management within this patient population. In particular, patients with Meth-APAH should be included in post-drug approval (ie, phase IV onward) trials, in separate Meth-APAH-only clinical trials, and/or as subgroup analyses. From the vantage point of the panel, inconsistent follow-up/poor compliance and poor therapy adherence associated with active methamphetamine use are challenges to including patients with Meth-APAH in clinical trials. Furthermore, the real or perceived lack of patient reliability and ongoing methamphetamine use/relapse interfering with interpretation of trial results are factors that impact the recruitment of patients with Meth-APAH to clinical trials.

## Discussion

This research explored expert opinions on the classification and treatment considerations for Meth-APAH using a modified Delphi panel methodology. The findings provide novel insight regarding the definition of Meth-APAH. The panel broadly agreed with the proposed definition of Meth-APAH: WHO group 1 PAH in a patient with history of methamphetamine use. However, the definition of Meth-APAH did not reach consensus because many panelists thought that the definition should also consider the cumulative exposure in duration and dosage of methamphetamine needed to cause Meth-APAH. Unfortunately, this threshold cannot be established in the absence of clear data on the topic. An overarching theme that emerged was the heterogeneity of the definition of Meth-APAH that clinicians use in their everyday practice. This reiterates the need for a standardized clinical definition of Meth-APAH to facilitate identification of these patients and provision of optimal treatment strategies.

Multiple characteristics of Meth-APAH were agreed on. However, the panel thought that low SES is not an appropriate way to characterize Meth-APAH because individuals who use methamphetamine can belong to any SES. Although, Kolaitis et al^13^ observed that patients with Meth-APAH typically have a poorer SES than patients with IPAH, the assumption that every patient with Meth-APAH is of a lower SES could lead to health care provider bias and may impact the care received by these patients. Developing clear clinical characteristics of Meth-APAH could reduce the focus on stigmatized characteristics (eg, SES) to improve the identification and management of patients with Meth-APAH.

The panel agreed that all patients should be asked about their history of methamphetamine use. Moreover, despite agreement across assessments that distinguish between Meth-APAH and other forms of PAH, the panel did not agree that there was a specific symptom or set of symptoms that defined Meth-APAH relative to other forms of PAH, given the broader heterogeneity of the PAH patient population. Universal Meth-APAH screening for patients with PAH was recommended; however, regional consent laws may prevent universal screening, and patients may not disclose their methamphetamine use due to the associated negative connotations.

Methamphetamine cessation should be a treatment goal for patients who actively use methamphetamine; however, cessation alone was thought to be insufficient to prevent disease progression, and the panel agreed that PAH-specific pharmacotherapy was an essential component of management. All patients with Meth-APAH can and should receive oral therapy in a manner similar to patients with IPAH regardless of methamphetamine user type, with no Meth-APAH-specific barriers identified to the adoption of oral combination therapies. This is supported by case reports of 3 patients with Meth-APAH, all of whom experienced clinical improvement after oral combination therapy^23^; however, additional literature is limited. Barriers that may impact the safety and practicality of treatment choices include methamphetamine user type, route of administration, and poor adherence to therapy. The panel agreed that methamphetamine user types were not well defined (eg, recent and long-term abstinence). This could make it harder to determine standardized methods for treating different user types. Furthermore, although rare, IV methamphetamine use was thought to be a particularly strong barrier to the use of IV or SC PAH therapies (eg, prostacyclin analogs), but that methamphetamine use status alone did not preclude use of IV or SC PAH therapies among other individuals with methamphetamine use disorder.

A holistic approach and financial aid are potentially useful strategies to manage nonadherence to therapy in Meth-APAH. Additionally, strategies such as contingency management and additional inpatient/rehabilitation support should be provided as management options for Meth-APAH. However, there are logistical issues and a lack of resources that may prevent all patients having access to these support strategies. Additional barriers that can impact the implementation of these support strategies include cost, transport, and access to care.^24^ Although this research was focused on the treatment of PAH associated with methamphetamine use, it is also important to consider structured, evidence-based addiction care as a method for reducing methamphetamine use. The potential role of a qualified psychiatrist, addiction medicine specialist, or counselor in Meth-APAH treatment is an area that requires further exploration.

Currently, there are few Meth-APAH-specific clinical trials and Meth-APAH-specific subgroup analyses.^17^ Due to the exclusion of patients with Meth-APAH from randomized controlled trials, there is currently a paucity of literature regarding the appropriate management of this population.^23^ The panel achieved consensus that patients with Meth-APAH should be included in clinical trials to generate information regarding treatment and disease management within this patient population. However, there may be challenges to including patients with Meth-APAH in randomized controlled trials, such as loss to follow-up/poor compliance and poor adherence associated with active methamphetamine use. Furthermore, factors that impact the recruitment of patients with Meth-APAH include their real or perceived ongoing methamphetamine use/relapses interfering with interpretation of trial results, and protocol-imposed restrictions regarding inclusion and exclusion criteria. Despite these challenges, including patients with Meth-APAH in clinical trials is necessary to aid in the definition and characterization of this distinct PAH phenotype.

This modified Delphi study had a few limitations, for example, we included only US-based experts, limiting the generalizability of the results. Additionally, regional differences may be present within the United States due to the varying prevalence of methamphetamine use in different states and the impact of differences in approach to drug screening; because this study recruited physicians with experience in managing Meth-APAH, many participants were based in Western United States. Furthermore, despite recruitment being conducted on an ongoing basis until quotas were filled, there was a large proportion of physicians who were not from Pulmonary Hypertension Association-designated Center of Comprehensive Care and who were practicing for only 3 to 5 years. Therefore, this may have biased the results. Furthermore, the panel included only 1 cardiologist with most panelists (n = 11) being pulmonologists; therefore, the perspectives given may differ from that of cardiology specialists. Despite the use of a moderator and chat function during the consensus meeting, different personalities and willingness to vocalize opinions may have biased the results. Furthermore, the questionnaires were completed in an unregulated environment and thus relied on the assumption that answers were considered and answered as accurately as possible.

## Interpretation

The panelists broadly agreed with the following definition of Meth-APAH: WHO group 1 PAH in a patient with history of methamphetamine use; however, the definition of Meth-APAH did not reach consensus.

A better understanding of the cumulative exposure to methamphetamine including duration, dosage, and time from first use needed to cause Meth-APAH limits a more specific and clinically helpful definition. Consensus was reached that all patients with PAH should be screened for Meth-APAH. Furthermore, all patients with Meth-APAH should be treated according to guidelines applicable to other forms of PAH including double combination therapies, with consideration to additional agents, including parenteral prostacyclin analogs in carefully selected patients. The characterization of Meth-APAH established during this study warrants further research and may help facilitate the identification, treatment, and management of patients with Meth-APAH to improve clinical outcomes.

## Funding/Support

This study was funded by Johnson & Johnson.

## Financial/Nonfinancial Disclosures

The authors have reported to *CHEST Pulmonary* the following: R. Z. declares grants to his institution from Gossamer Bio, Merck, United Therapeutics, and Janssen; consulting fees from Gossamer Bio, Morphogen IX, Merck, and Aerovate; patents planned, issued, or pending for FK506 for treatment of pulmonary hypertension; participation on a data safety monitoring board or advisory board for Aerovate; and stock options in REVIVA. P. J. L. has received support for attending meetings and/or travel, and received support for data access, analysis, and writing from Janssen/Actelion Pharmaceuticals; has received grants from NIH/NHLBI, Bayer Pharmaceuticals (PHAB Award) Mentor, and the Cystic Fibrosis Foundation Therapeutic Development Network, and consulting fees from Sumitimo Pharma; and has participated on a data safety monitoring board or advisory board for NHLBI and in a leadership or fiduciary role for Team PHenomenal Hope and the Pulmonary Hypertension Association Registry. T. S. has received consultancy fees from Janssen Scientific Affairs, LLC. J. K. has served on the speaker’s bureau for Bayer Pharmaceuticals, United Therapeutics, Merck, Gossamer Bio, and J&J Innovative Medicine; has served as a consultant for United Therapeutics and J&J Innovative Medicine; and has participated in clinical research funded by United Therapeutics, Bayer, Janssen, Phase Bio, Gossamer Bio, and Acceleron. D. L., A. D. A., G. D., and M. Sandros are employees of Johnson & Johnson. M. Stone, D. B., R. P., A. E., L. P., and H. S. are employees of Adelphi Values PROVE, who were contracted by Johnson & Johnson to conduct this research. N. H. K. has served as consultant and/or speaker for J&J, Bayer, Merck, United Therapeutics, Gossamer Bio, and Pulnovo.

## Supplementary Data

e-Online Data
