# Supplementary material for: Expert Consensus to Explore the Definition and Characterization of Methamphetamine-Associated Pulmonary Arterial Hypertension and Key Treatment Considerations
Source: CHEST Pulm. 2025 Dec 31;4(2):100232. doi: 10.1016/j.chpulm.2025.100232 (PMC13418898; doi:10.1016/j.chpulm.2025.100232)
Supplement: e-Online Data [file mmc1.docx]

# Supplementary material

Table S1. All statements that achieved a consensus in agreement throughout the three modified‑Delphi rounds

| **#** | **Question/statement** | **Consensus (% agreed with the statement)** | **Mean agreement score  (range: 1–9)** | **Stage consensus reached** |
| --- | --- | --- | --- | --- |
|  | **Defining methamphetamine-associated PAH (Meth-APAH)** | | | |
| 1 | Physicians would expect among patients presenting with Meth‑APAH to be aged between 21 and 60 | 21–30 years: 92%  31–40 years: 100%  41–50 years: 92%  51–60 years: 83% | N/A | 2 |
| 2 | Meth-APAH can occur in any age group, but it is most seen in those aged 21–60 years | 100% (n=10) | N/A | 3 |
| 3 | Meth-APAH can occur in patients of any socioeconomic status | 100% (n=10) | N/A |  |
| 4 | There should be more intensified (more frequent or consistent) screening between those aged 31 and 50 | 86% | N/A | 2 |
|  | **Meth-APAH versus idiopathic PAH** | | | |
| 5 | Frequency of dosing is a treatment-related factor that plays a role in treatment choice for patients with Meth-APAH | 83% | 8 | 1 |
| 6 | Route of administration is a treatment-related factor that plays a role in treatment choice for patients with Meth-APAH | 92% | 9 |  |
| 7 | Clinical trial results is a treatment-related factor that plays a role in treatment choice for patients with Meth-APAH | 92% | 8 |  |
| 8 | Meth-APAH and idiopathic PAH be considered different regarding patient prognosis | 83% | 8 | 2 |
| 9 | Meth-APAH and idiopathic PAH be considered different regarding social support | 92% | 8.5 |  |
| 10 | Meth-APAH and idiopathic PAH be considered different regarding withdrawal signs and symptoms | 92% | 8 |  |
| 11 | Meth-APAH differs from idiopathic PAH (IPAH) in disease course | 100% (n=10) | N/A | 3 |
| 12 | Meth-APAH differs from IPAH in patient management considerations | 100% (n=10) | N/A |  |
| 13 | Meth-APAH differs from IPAH in treatment considerations | 100% (n=10) | N/A |  |
|  | **Diagnosis of patients with Meth-APAH** | | | |
| 14 | Discussion of drug history distinguishes patients with Meth-APAH from patients from other forms of PAH | 100% | 9 | 1 |
| 15 | Previous methamphetamine use distinguishes patients with Meth‑APAH from patients from other forms of PAH | 92% | 9 |  |
| 16 | Urine screening/toxicology distinguishes patients with Meth-APAH from patients from other forms of PAH | 83% | N/A |  |
| 17 | All physicians inquire about/explore the potential for methamphetamine use/history for all referrals/new cases of PAH | 100% | N/A |  |
| 18 | All patients presenting with PAH should be screened for Meth‑APAH | 100% (n=10) | N/A | 3 |
|  | **Barriers to identifying patients with Meth-APAH and methamphetamine user-types** | | | |
| 19 | Patients with disclosed or suspected methamphetamine use should be drug tested as part of routine disease management | 100% | 8 | 1 |
| 20 | The current user (use within past month) methamphetamine user type is likely to impact successful Meth-APAH outcomes | 83% | 9 |  |
| 21 | Access to a range of/advanced therapies is impacted by methamphetamine user-type | 100% | 9 | 2 |
| 22 | Compliance (to therapy) is impacted by methamphetamine user-type | 100% | 9 |  |
| 23 | Treatment escalation is impacted by methamphetamine user-type | 92% | 9 |  |
| 24 | The timeframes that define different methamphetamine user types are not well established and lack precision | 100% (n=10) | N/A | 3 |
| 25 | Left heart disease is a comorbidity that can cause challenges in identifying patients with Meth‑APAH | 100% (n=10) | N/A |  |
| 26 | HIV is a comorbidity that can cause challenges in identifying patients with Meth‑APAH | 83% | 7 | 2 |
| 27 | The loss to follow-up is a key challenge to managing patients with Meth-APAH | 100% | 8.5 |  |
| 28 | Lack of stable housing is a barrier to identifying patients with Meth-APAH and engaging them in care | 100% | 8 |  |
| 29 | Lack of familial and caregiver support is a barrier to identifying patients with Meth‑APAH and engaging them in care | 92% | 8.5 |  |
| 30 | Patient engagement with care is a barrier to identifying patients with Meth APAH and engaging them in care | 92% | 8 |  |
| 31 | Access to transportation is a barrier to identifying patients with Meth APAH and engaging them in care | 92% | 7 |  |
| 32 | Socioeconomic status is a barrier to identifying patients with Meth APAH and engaging them in care | 83% | 8 |  |
| 33 | 6-minute walking test (6MWT) does not distinguish patients with Meth-APAH from patients from other forms of PAH | 92% | N/A |  |
| 34 | Cardiac magnetic resonance imaging (cMRI) does not distinguish patients with Meth‑APAH from patients from other forms of PAH | 92% | N/A |  |
| 35 | All patients presenting with PAH should be screened for Meth-APAH | 92% | 9 |  |
| 36 | Physicians verbally ask the patient if they are using methamphetamine | 100% | N/A |  |
| 37 | Physicians would still consider right heart catheterization (RHC) in a patient who is a known active user of methamphetamine | 92% | N/A |  |
| 38 | Support from family or caregivers should be very involved in the management of patients with Meth-APAH | 92% | 9 |  |
| 39 | Clinician familiarity with Meth-APAH is a challenge in identifying and engaging patients with Meth-APAH | 100% (n=10) | N/A | 3 |
|  | **Barriers to treatment of patients with Meth-APAH** | | | |
| 40 | Intravenous injection therapies require additional considerations for patients with Meth-APAH | 100% | N/A | 2 |
| 41 | Subcutaneous injection therapies require additional considerations for patients with Meth-APAH | 83% | N/A |  |
| 42 | Physicians are likely to prescribe subcutaneous injection therapies when the patient with Meth-APAH is compliant and achieved sobriety | 83% | 8 |  |
| 43 | Use of intravenous methamphetamine impacts the use of intravenous (IV) and/or subcutaneous (SC) therapies | 100% (n=10) | N/A | 3 |
| 44 | Whether injection therapies are administered should be dependent on safety and practicality rather than methamphetamine use status | 100% (n=10) | N/A |  |
| 45 | Treatment escalation is a barrier to treatment of patients with Meth‑APAH | 83% | 7 | 2 |
| 46 | The perception in the medical community that a patient with Meth‑APAH is not eligible to be treated is a barrier to treatment of patients with Meth-APAH | 83% | 8 |  |
| 47 | Route of administration is a barrier to treatment of patients with Meth-APAH | 92% | 8 |  |
| 48 | Delay/failure to refer patients to a specialist is a barrier to treatment for patients with Meth APAH | 100% (n=10) | N/A | 3 |
| 49 | Adverse event management is a barrier to treatment for patients with Meth-APAH | 100% (n=10) | N/A |  |
| 50 | Methamphetamine cessation should be one of the main treatment goals for patients with Meth APAH | 100% (n=10) | N/A |  |
|  | **Treatment choice/implications for patients with Meth-APAH** | | | |
| 51 | Methamphetamine use status is a disease-related factor that plays a role in treatment choice for patients with Meth-APAH | 83% | 8 | 1 |
| 52 | Presence of right-side heart failure is a disease-related factor that plays a role in treatment choice for patients with Meth-APAH | 83% | 9 |  |
| 53 | Registry to Evaluate Early and Long-term PAH Disease Management (REVEAL) risk score is a disease-related factor that plays a role in treatment choice for patients with Meth-APAH | 83% | 8 |  |
| 54 | Risk assessment or risk stratification is a disease-related factor that plays a role in treatment choice for patients with Meth-APAH | 83% | 8 |  |
| 55 | Symptom severity at diagnosis is a disease-related factor that plays a role in treatment choice for patients with Meth-APAH | 83% | 8 |  |
| 56 | Patient's compliance with healthcare team is a patient-related factor that plays a role in treatment choice for patients with Meth-APAH | 92% | 8.5 |  |
| 57 | Ability to communicate/interact with the healthcare team is a patient-related factor that plays a role in treatment choice for patients with Meth-APAH | 92% | 8 |  |
| 58 | Patient’s social/caregiver support/friends/family is a patient-related factor that plays a role in treatment choice for patients with Meth-APAH | 83% | 8 |  |
| 59 | Stable housing/stable employment is a patient-related factor that plays a role in treatment choice for patients with Meth-APAH | 92% | 8 |  |
| 60 | Mental health/psychiatric conditions are patient-related factors that play a role in treatment choice for patients with Meth-APAH | 92% | 7.5 | 2 |
| 61 | Comorbidities are disease-related factors that play a strong role in treatment choice for patients with Meth-APAH | 92% | 7 |  |
| 62 | Side effect profile is a treatment-related factor that plays a role in treatment choice for patients with Meth-APAH | 92% | 7 |  |
| 63 | Treatment compliance is a treatment-related factor that plays a role in treatment choice for patients with Meth-APAH | 100% | 8 |  |
| 64 | Geographical access to care is a treatment-related factor that plays a role in treatment choice for patients with Meth-APAH | 83% | 7 |  |
| 65 | Patients who exhibit a vasoreactivity response would be offered different treatment options for Meth-APAH | 100% (n=10) | N/A | 3 |
| 66 | Treatment-related factors e.g., HIV and CHD, would impact treatment choice for patients with Meth-APAH | 100% (n=10) | N/A |  |
| 67 | Treatment-related factors e.g., ADHD, would have no impact on treatment choice for patients with Meth-APAH | 100% (n=10) | N/A |  |
| 68 | Clinical trial results impact treatment choice for patients with Meth-APAH | 100% (n=10) | N/A |  |
| 69 | Treatment approach (monotherapy/combination therapy) impacts treatment choice for patients with Meth-APAH | 100% (n=10) | N/A |  |
| 70 | Social support impacts treatment choice for patients with Meth-APAH | 100% (n=10) | N/A |  |
| 71 | A patient’s treatment adherence history impacts treatment choice for Meth-APAH | 100% (n=10) | N/A |  |
| 72 | Participation in a substance abuse program impacts treatment choice for Meth-APAH | 100% (n=10) | N/A |  |
| 73 | There is no barrier to combination therapies (i.e., all patients with Meth-APAH can receive combination therapy) | 100% (n=10) | N/A |  |
| 74 | All physicians would treat a patient with Meth-APAH with PH medication even if they are actively using methamphetamine | 100% | N/A | 1 |
| 75 | The diagnosis of Meth-APAH vs other forms of PAH plays a factor when deciding on treatment of Meth-APAH (assuming disease severity, contraindications, comorbidities, and/or socioeconomic status are comparable)? | 92% | N/A |  |
| 76 | The approach to the route of administration changes in patients that have Meth‑APAH vs other forms of PAH (i.e., inhaled, intravenous, oral, subcutaneous) | 92% | N/A |  |
| 77 | Patient failure to attend appointments and receive treatment is a barrier to treatment of patients with Meth-APAH | 92% | 8 |  |
| 78 | Patient decision to cease treatment is a barrier to treatment of patients with Meth‑APAH | 83% | 8 |  |
| 79 | Patient failure to comply with instructions regarding their treatment is a barrier to treatment of patients with Meth-APAH | 83% | 8 |  |
| 80 | Insurance/cost barriers is a barrier to treatment of patients with Meth-APAH | 92% | 8 |  |
| 81 | Patients with Meth-APAH should be treated with PAH therapies alongside methamphetamine discontinuation | 100% | 9 |  |
| 82 | Patients with Meth-APAH should be considered for PAH therapies even if methamphetamine has not been discontinued | 100% | 9 |  |
| 83 | A patient with Meth-APAH should be on combination therapy if compliant to therapy, but still using methamphetamine | 100% | 8 | 2 |
| 84 | If oral medications that combine different pathways were available to reduce pill count, physicians would be very likely to prescribe them to patients with Meth‑APAH | 92% | 8.5 |  |
| 85 | The prescription of one (combination) tablet would affect patients with Meth-APAH and their treatment by improving adherence | 83% | 8 |  |
| 86 | The prescription of oral medications that combine different pathways would result in improved persistence | 100% (n=10) | N/A | 3 |
| 87 | The prescription of oral medications that combine different pathways would result in improved quality of life | 100% (n=10) | N/A |  |
| 88 | The prescription of oral medications that combine different pathways would result in improved health outcomes | 100% (n=10) | N/A |  |
|  | **Treatment non-adherence in patients with Meth-APAH** | | | |
| 89 | Enrolling in a substance abuse program is a useful strategy to manage treatment non‑adherence in patients with Meth-APAH | 83% | 8 | 1 |
| 90 | Dissociating patients from high-risk environment is a useful strategy to manage treatment non‑adherence in patients with Meth-APAH | 92% | 8 |  |
| 91 | Healthcare provider attitudes, i.e., conveying non-judgmental, empathic verbal and nonverbal behaviors is a useful strategy to manage treatment non‑adherence in patients with Meth-APAH | 100% | 8.5 |  |
| 92 | Patient openness to discussing their substance use is a useful strategy to manage treatment non‑adherence in patients with Meth-APAH | 92% | 8 |  |
| 93 | Patient understanding of the association between methamphetamine use and PAH is a useful strategy to manage treatment non‑adherence in patients with Meth-APAH | 83% | 9 |  |
| 94 | Social worker support is a useful strategy to manage treatment non‑adherence in patients with Meth-APAH | 83% | 8 |  |
| 95 | Healthcare provider familiarity with the terminology associated with methamphetamine use, including the terms used for the drug itself is a useful strategy to manage treatment non‑adherence in patients with Meth-APAH | 100% | 7 | 2 |
| 96 | The holistic approach and financial aid are useful strategies for managing non‑adherence to therapy in patients with Meth-APAH | 100% (n=10) | N/A | 3 |
|  | **Meth-APAH in clinical trials** | | | |
| 97 | The real or perceived lack of reliability complying with trial recruiters’ instructions is a factor that impacts recruitment of patients with Meth-APAH in clinical trials | 92% | 8.5 | 1 |
| 98 | The real or perceived ongoing methamphetamine use/relapse interfering with interpretation of trial results is a factor that impacts recruitment of patients with Meth-APAH in clinical trials | 83% | 8 |  |
| 99 | Loss to follow-up/poor compliance and adherence associated with active methamphetamine use is a challenge to including patients with Meth-APAH in clinical trials | 92% | 8.5 | 2 |
| 100 | The real or perceived views of clinical trial recruiters is a challenge to including patients with Meth-APAH in clinical trials | 83% | 7 |  |
| 101 | Patients with Meth-APAH should be included in post-drug approval clinical trials (i.e., Phase IV onwards) | 100% | 8.5 |  |
| 102 | Patients with Meth-APAH should be included in separate Meth-APAH only clinical trials or as subgroup analyses | 100% | 8 |  |
| 103 | Patients with Meth-APAH should be included as a part of clinical trials in order to generate information regarding treatment and disease management within this treatment population | 100% (n=10) | N/A | 3 |
| 104 | Protocol imposed restrictions/inclusion and exclusion criteria are challenges to recruiting patients with Meth-APAH to clinical trials | 100% (n=10) | N/A |  |
| 105 | Deciding which stages of clinical trials that patients with Meth-APAH can be included in is dependent on the definition of Meth-APAH | 100% (n=10) | N/A |  |
|  | **Management of patients with Meth-APAH** | | | |
| 106 | Drug rehabilitation support should be involved in the management of Meth-APAH patients | 83% | 9 | 1 |
| 107 | Patient education should be involved in the management of Meth-APAH patients | 100% | 9 |  |
| 108 | Shared clinical decision-making should be involved in the management of Meth‑APAH patients | 100% | 9 |  |
| 109 | Social workers should be involved in the management of Meth-APAH patients | 83% | 9 |  |
| 110 | Additional drug rehabilitation support should be provided as a management option for patients with Meth-APAH | 100% | N/A |  |
| 111 | Additional inpatient/rehab support would be useful for patients with Meth-APAH | 100% (n=10) | N/A | 3 |
| 112 | Contingency management support would be useful for patients with Meth-APAH | 100% (n=10) | N/A |  |
| 113 | Incentives would be useful for patients with Meth-APAH | 100% (n=10) | N/A |  |

Stage consensus reached: 1: first-round survey; 2: second-round survey; 3: consensus meeting.

N/A = not applicable.
